# Innovative Diagnostic Solutions in Hemostasis

**DOI:** 10.3390/diagnostics14222521

**Published:** 2024-11-11

**Authors:** Emmanuel J. Favaloro, Leonardo Pasalic

**Affiliations:** 1 Haematology Department, Institute of Clinical Pathology and Medical Research (ICPMR), Sydney Centres for Thrombosis and Haemostasis, Westmead Hospital, Westmead, NSW 2145, Australia; leonardo.pasalic@health.nsw.gov.au; 2 School of Dentistry and Medical Sciences, Faculty of Science and Health, Charles Sturt University, Wagga Wagga, NSW 2650, Australia; 3 School of Medical Sciences, Faculty of Medicine and Health, University of Sydney, Westmead Hospital, Westmead, NSW 2145, Australia; 4 Westmead Clinical School, University of Sydney, Westmead, NSW 2145, Australia

**Keywords:** hemostasis diagnostics, therapeutic monitoring, instrumentation, innovation

## Abstract

Hemostasis describes the process of blood clotting homeostasis. Hemostasis reflects a balance of procoagulant and anticoagulant mechanisms that aim to prevent both bleeding and thrombosis. If hemostasis is disrupted, and bleeding or thrombosis occur, then laboratory testing may ensue to either diagnose the reason for bleeding or thrombosis, or to manage patients under therapy or treatment for bleeding or thrombosis. A wide range of tests of hemostasis are available to laboratories and to clinicians, from routine coagulation assays to specialized hemostasis assays and platelet function. In the current narrative review, we highlight some of the history of innovative diagnostic solutions, such as the integration of chemiluminescence and flow cytometry in the hemostasis diagnostic armamentarium, as well as providing a glimpse to the possible future of diagnostic hemostasis testing. Future directions include the potential for artificial intelligence in diagnostics, the development of more global test systems that can assess both primary and secondary hemostasis, and several innovations to enable the ongoing evolution of therapies to rebalance hemostasis and requiring precise monitoring. This review underscores the ongoing need for innovation to enhance the diagnostic landscape of hemostasis, ensuring better patient outcomes through more accurate and efficient diagnostic methods.

## 1. Introduction to Hemostasis

**Figure 1:**
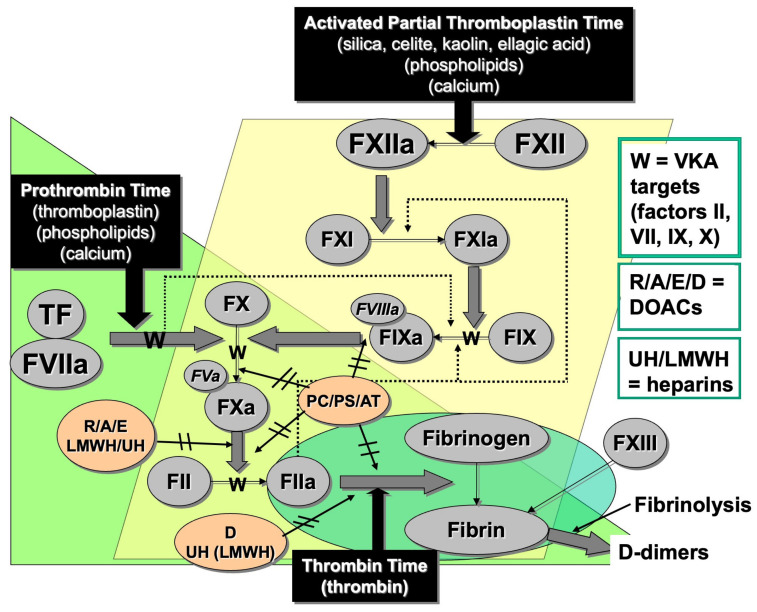
An overview of the main secondary hemostasis coagulation pathways, and also corresponding routine coagulation assays. Also shown are relevant anticoagulant points, both physiological and clinical. Protein C (PC), protein S (PS), and antithrombin (AT) are natural anticoagulants that act primarily on FV and FVIII (PS/PC) or on thrombin (FIIa [AT]). The classical clinical anticoagulants are warfarin (W) and alternative vitamin K antagonists (VKAs) affecting FII, FVII, FIX, and FX, and the heparins (unfractionated heparin [UH] and low molecular weight heparin [LMWH] affecting FXa (both UH and LMWH) and thrombin (FIIa; mostly UH). More recent clinical anticoagulants comprise the direct oral anticoagulants (DOACs), currently including the anti-FIIa agent dabigatran (D), and the anti-FXa agents rivaroxaban (R), apixaban (A), and edoxaban (E).

Hemostasis describes the process of blood clotting homeostasis. Hemostasis involves a wide range of plasma proteins and blood cell components, especially platelets, and the vascular endothelium [1,2]. The plasma proteins include so-called coagulation factors, generally identified by Roman numerals, as well as adhesion proteins, and proteins involved in fibrinolysis. The coagulation proteins are often grouped into coagulation pathways, including terms, such as ‘coagulation cascade’, ‘intrinsic pathway’ (or ‘contact factor pathway’), ‘extrinsic pathway’ (or ‘tissue factor pathway’), and ‘common pathway’ (Figure 1). The coagulation process involves a process of sequential enzymic activation of coagulation proteins that leads to the eventual formation of a ‘clot’, largely composed of the soluble coagulation protein fibrinogen, after its conversion to an insoluble fibrin. These processes can be evaluated in hemostasis testing laboratories, either using routine coagulation tests or specialized hemostasis testing (Table 1 and Table 2) [1]. For example, the prothrombin time (PT) assay starts at factor (F) VII (FVII), enters the common pathway at FX, and ends at fibrin formation, which is typically detected by an automated hemostasis analyzer (Figure 1). Instead, the activated partial thromboplastin time (APTT) assay starts at FXII, but after a few sequential factor activations it also enters the common pathway at FX, and also terminates with fibrin formation, again typically detected by automated hemostasis analyzers (Figure 1).

Additional routine assays assessable by hemostasis laboratories within this pathway are the thrombin time (TT) assay and fibrinogen assays (Figure 1 and Table 1). All these routine assays are performed using clot formation detection and can be assessed with standard automated hemostasis analyzers. The fibrinogen level is most usually assessed as a so-called functional von Clauss assay, named after its inventor [3]. It is also possible to perform assays to identify the level of individual clotting factors, which are otherwise involved in secondary hemostasis (Table 2). These are usually measured using so-called one-stage assays that reflect modifications of the PT (typically for FVII, FX, FII, FV) or APTT (FVIII, FIX, FXI, FXII) [4]. An additional factor that is important in hemostasis but not measured in the same way as the other factors is FXIII (Figure 1) [5]. FXIII acts on any forming fibrin to stabilize the fibrin clot. Because of their relative test complexity and less frequent performance, factor assays are usually performed in more specialized hemostasis laboratories. Collectively, the clotting factors are considered ‘procoagulant’ proteins, as they promote coagulation. A deficiency of clotting factors, except for FXII, may, therefore, lead to bleeding, and an excess may, in some cases, lead to thrombosis [6]. To provide the balance for hemostasis, there are several physiological ‘anticoagulants’ that aim to dampen excess coagulation. These include antithrombin, protein C, and protein S [1,6]. These act on different aspects of the coagulation pathway and are typically measured (if needed) by specialized hemostasis laboratories. Deficiencies of these physiological anticoagulants can lead to thrombosis [6].

In addition to secondary hemostasis, additional adhesion proteins and cellular elements, especially platelets, contribute to the process of primary hemostasis [1,7]. The main adhesion protein in blood involved in hemostasis is called von Willebrand factor (VWF). Upon tissue injury, VWF binds to the damaged tissue, especially another protein called collagen, which activates VWF to expose the platelet receptor binding site on VWF [7]. VWF can then bind to platelets via the glycoprotein Ib (GPIb) receptor, causing platelet activation and leading to platelet aggregation and the formation of a platelet plug. In vivo, primary and secondary hemostasis act together to form a stable platelet/fibrin plug. VWF has additional hemostatic functions, including binding FVIII, thereby stabilizing and protecting FVIII from degradation, as well as delivering this important clotting factor to sites of injury to promote secondary hemostasis. Tests for VWF and platelet function are typically performed by specialized hemostasis laboratories (Table 2) [1].

After clot formation, the fibrinolysis pathway is activated to prevent excessive clot formation, to dissolve the clot, and to permit tissue repair [8,9]. The level of active in vivo fibrinolysis can be measured by a blood test called D-dimer, since this is a specific breakdown product arising from fibrin formation and breakdown. D-dimer testing usually forms part of routine coagulation (Table 1) [1]. The composite processes of clot formation and breakdown are considered parts of the secondary hemostasis pathways, although since in vivo primary and secondary hemostasis are intertwined, the links to primary hemostasis are also important.

## 2. When Hemostasis Fails—Anticoagulant and Procoagulant Therapy

**Figure 2:**
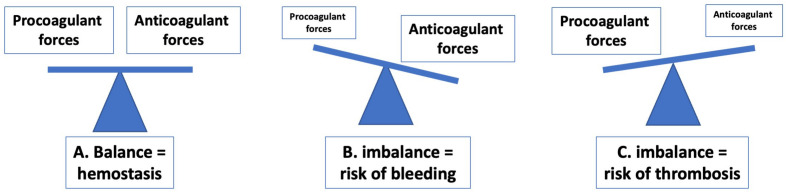
A pictorial representation of hemostasis in balance (**A**) (left), with procoagulant and anticoagulant forces in balance. When procoagulant forces are decreased (e.g., due to lack of clotting factors [e.g., hemophilia]), hemostasis is not in balance, and this can lead to bleeding (**B**) (middle). It is possible to increase the procoagulant forces (e.g., factor replacement in hemophilia) to rebalance hemostasis (i.e., drive hemostasis towards rebalance as in (**A**)). When anticoagulant forces are decreased (e.g., due to a deficiency in PC, PS, or AT), hemostasis is not in balance, and this can lead to thrombosis (**C**) (right). It is possible to increase the anticoagulant forces (e.g., by use of clinical anticoagulants) to rebalance hemostasis (i.e., to drive hemostasis towards rebalance as in (**A**)). In addition, an imbalance towards bleeding can also occur if anticoagulant forces are in excess (e.g., too much clinical anticoagulant applied), and an imbalance towards thrombosis can also occur if procoagulant forces are in excess (e.g., an excess of clotting factors are present). The hemostasis laboratory can assess elements of this hemostasis balance using a wide repertoire of routine coagulation (Table 1) and specialized hemostasis assays (Table 2 and Table 3).

As noted previously, hemostasis represents a balance of pro- and anticoagulant forces aiming to prevent bleeding and thrombosis (Figure 2). Insufficiency in procoagulant factors (e.g., deficiency of clotting factors [except FXII], VWF or platelets, or their reduced activity) can lead to bleeding (e.g., hemophilia, von Willebrand disease [VWD]) [10,11], whereas insufficiency in anticoagulant factors (e.g., deficiency of PC, PS, or AT), or an excess of procoagulant factors can lead to thrombosis [6]. It is possible to prevent or treat either bleeding or thrombosis by ‘replacing’ the missing or defective factors or otherwise by rebalancing hemostasis. For example, in patients deficient in FVIII (i.e., with hemophilia A), therapy to treat or prevent bleeding may include the replacement of FVIII, or use of FVIII bypassing therapy, or other means to rebalance hemostasis [10]. Similarly, in patients with VWD, bleeding can be treated or prevented using VWF concentrates or other agents [11,12]. In patients with thrombosis, or a tendency towards thrombosis, these can be treated with various clinical anticoagulants or ‘anti-thrombotics’ [13]. Of relevance, there have been extraordinary advances in such therapies over the past few years (discussed later). In addition, it is also possible to monitor most of the above therapy using laboratory testing.

## 3. When Hemostasis Is Challenged

Hemostasis is normally in balance in otherwise normal individuals (Figure 2). However, hemostasis can be challenged by a range of factors, including trauma, infection, poor nutrition, disease, cancer, and surgery. Although this topic in itself is worthy of a comprehensive review, it may suffice here just to provide some pertinent examples. Trauma may occur as a result of any accidental injury and can range from mild to severe or life threatening. In particular, major trauma can lead to a substantial loss of blood, as well as ongoing challenges to repair the trauma [14,15]. In general, trauma leads to an imbalance of hemostasis towards bleeding, primarily due to a loss of procoagulants (Figure 2B). This will also lead to derangements in most tests of hemostasis (Table 1, Table 2 and Table 3). In general, the aim of clinicians managing trauma patients is the replacement of the lost procoagulants, and these may include fibrinogen, clotting factors, and platelets. Trauma therapy can in part be guided by laboratory testing that aims to assess the degree of normalization.

That infection can lead to hemostasis derangement is perhaps best illustrated by the example of the contemporary pandemic/endemic that we call COVID-19 (coronavirus disease 2019). Although a viral disease, COVID-19 causes derangement of many hemostasis pathways, reflecting the activation of hemostasis and subsequent thrombosis risk [16,17,18]. COVID-19 can affect many hemostasis pathways and leads to abnormalities in many hemostasis tests, from routine assays, D-dimer, and platelet counts, to VWF, and a reflected increase in autoimmune markers, including, for example, as measured by LA testing.

Diet can affect hemostasis in many ways [19,20,21]. Perhaps the most well-known is the importance of dietary vitamin K, especially in patients on VKA therapy, but also in patients severely deficient in vitamin K, since this will reduce the functionality of the vitamin K-dependent factors (i.e., FII, FVII, FIX, FX). However, many other dietary components can affect hemostasis, including vitamin C, and platelet function (e.g., fish oils, chocolate, and garlic).

## 4. A Brief Review of Past Innovative Diagnostic Solutions in Hemostasis

The history of hemostasis testing is rich in innovation [2]. In large part, hemostasis researchers and laboratories developed tests to improve on existing methods. For example, one of the historical ‘classical’ anticoagulants used to treat or prevent thrombosis is warfarin, which is a vitamin K antagonist (VKA) that interferes with production of selective fully functional clotting factors (namely, FII, FVII, FIX and FX) (Figure 1). Because patients have a variable therapeutic response to the action of warfarin, which is further affected by diet and other drug interactions, the VKA effect on coagulation in individual patients needs to be monitored by laboratory testing [19]. Too much warfarin can lead to bleeding, and too little warfarin can lead to thrombosis. The original test used for this purpose was the PT, but different PT reagents have different degrees of sensitivity to the effects of warfarin, due to the variable sensitivities to FII, FVII, FIX, and FX. Thus, the PT developed into a test called the INR (international normalized ratio), which adjusted for the variable reagent sensitivity. The INR is actually a mathematical formula = (PT/MNPT)^ISI^, where PT is the patient’s PT, MNPT is the mean normal PT, and ISI is the international sensitivity index, and represents a correction factor for the reagent/instrument variation). The INR represented a major advance in warfarin therapy monitoring and can also be performed on point-of-care (POC) instruments [22].

The APTT is also a relative advance of the historically used PTT, which represented a more variable non-activated form of the APTT. The APTT is useful to assess levels of clotting factors, and it can also be used to monitor UH therapy and as a test for investigation of the lupus anticoagulant (LA) [23,24].

For the assessment of factor activity, the one-stage clotting assay represents the original methodology, and is still the current most common method for laboratory assessment of clotting factors [4]. However, for hemophilia diagnosis, FVIII testing by this method sometimes provides inaccurate estimates of FVIII activity, as otherwise evidenced by the clinical assessment of hemophilia severity. Thus, the development of chromogenic assays that better matched clinical severity represented a major breakthrough in hemophilia diagnosis and management [25].

For VWD diagnosis, there has been a huge increase in the number of methods available to better diagnose VWD and its various subtypes (Table 3) [26,27,28,29,30]. Method developments have progressed from measurements of VWF level (‘antigen’; VWF:Ag) to VWF platelet GPIb binding function as an assessment by the ristocetin-induced platelet agglutination (RIPA) assay and the ristocetin cofactor (VWF:RCo) assay. Then, an assay to measure the ability of VWF to bind to damaged tissue, namely collagen, or the collagen-binding (VWF:CB) assay represented another advance [29], as did the assay that assesses the ability of VWF to bind to FVIII (i.e., the VWF:FVIII-binding assay) [30]. With each advance in VWD diagnostics came improvements in the diagnosis of VWD and refinement of its various subtypes to enable optimized VWD treatment therapy for affected patients.

## 5. Contemporary Innovative Diagnostic Solutions in Hemostasis

More recent innovations in hemostasis diagnostics are highlighted below.

### 5.1. INR Testing

Although the INR has improved the monitoring of VKA therapy, problems remain, since laboratories need to estimate and/or verify/validate two components of the INR, namely the MNPT and the ISI. This leads to wide variability of INR values for the same homogenous samples, as evidenced in external quality assessment (EQA) exercises [31]. In the past, the MNPT could be estimated using at least 20 normal individuals [31,32], but these results are increasingly difficult to source in contemporary ethical settings. Also, the use of different sets of 20 normal individuals leads to different MNPTs for the same PT reagents, thereby partly explaining INR variability [24]. Classically, estimation of the ISI requires an onerous method, using manual tilt method PTs of at least 20 normal individuals and 60 different samples from patients on stable warfarin therapy [32,33,34]. Alternative methods for generating both MNPT and ISI values use commercial calibration plasma sets [32], but these still generate variable MNPTs and ISI values [31], further explaining INR variability. We no longer use these classic methods to estimate and/or verify/validate MNPT and ISI values. Instead, we use a simple process of linear regression to compare replacement PT reagents against existing PT reagents, which has now involves a large network of laboratories, and helps maintain continued low bias and variability for INRs compared to peer median values, even after a complete change of reagents and instrumentation [34].

Several alternatives to use instead of PT or classical INR to monitor warfarin have also been proposed [35,36,37,38]. These include the FiiX PT method [35,36] and the chromogenic FX assay [37,38]. In particular, for the FiiX PT method, proponents of its use identify that whilst the antithrombotic effect of VKAs depend on controlled lowering of the activity of factors FII and FX, similar to that of other anticoagulants, reductions in FVII and FIX play a less important role [36]. Moreover, classical PT-INR-based monitoring is highly influenced by FVII, which has the shortest half-life of the vitamin K-dependent coagulation factors. Hence, variability in the anticoagulant effect of VKA may be partly secondary to an inherent flaw of the traditional monitoring test itself. The FiiX PT method reflects an assay that is only sensitive to reductions in FII and FX and is intended to stabilize the VKA effect. Similarly, the chromogenic FX assay [37,38] also specifically avoids the measurement of other VKA-affected factors.

### 5.2. Hemostasis Instrumentation

Hemostasis instrumentation has evolved over the past decades, as has, in part, been recently reviewed [2]. Some of the earlier semi-automated instruments required operators to change the direction and application of plastic tubing to permit a change of assays. Instruments have moved from optical clot detection to mechanical clot detection, and now some instruments have both as standard. The development of some alternate detection systems, such as chemiluminescence detection, permits analyte detection down to 0% [28,39]. Today, such systems can also be incorporated into standard hemostasis instruments, so that clot detection, turbimetric testing (e.g., latex immunoassays; LIA), chromogenic assays, and chemiluminescence immunoassays (CLIA) can all be performed on a single instrument. For example, one instrument from Stago Diagnostics (Asnières sur Seine, France) called the sthemO incorporates mechanical testing with optical testing, LIA, chromogenic assays, and chemiluminescence [40]. Along with such advances have come increasing accuracy and precision, as well as throughput. Our original hemostasis analyzer could perform a maximum of 24 tests/run of ~2 h [2]. Current analyzers can maintain test throughputs in the hundreds/hour. It is also possible to combine hemostasis instruments into an automated line, although we do not feel this to necessarily be an improvement, as it has the capacity to dumb-down hemostasis to simple chemistry, since chemistry systems tend to dominate such automations.

### 5.3. Clot Waveform Analysis

Somewhat linked to instrumentation is the ability to analyze the output from some instruments beyond the standard measures of clotting times in seconds. One form of analysis popular with certain researchers in that of clot waveform analysis (CWA), which analyzes the visual output of optical detection hemostasis analyzers [41,42,43,44,45]. This usually assesses the clot formed by the APTT, and has been used to assess various disorders, as well as some therapeutic interventions [41,42,43,44,45]. For example, elevated CWA values have been associated with hypercoagulability in venous thromboembolism, as well as acute myocardial infarction (AMI) [41]. As for other relevant examples, CWA has also been used to monitor recombinant FVII therapy [43], as well as to assess the in vitro effects of DOACs [44] and to evaluate FVIII concentrates [45].

### 5.4. VWF Testing

For VWD diagnosis and therapy monitoring, there has been a huge increase in the number of methods available to better diagnose VWD and its various subtypes (Table 3), as previously noted. Several new assays have been developed to measure platelet GPIb binding, and these have now largely replaced the classical VWF:RCo assay [26,27,28]. One assay uses recombinant GPIb (VWF:GPIbR) with added ristocetin and can be either performed as a latex agglutination assay on a standard hemostasis analyzer [46], or else by using magnetic beads and CLIA technology [39]. Another assay uses recombinant mutated GPIb (VWF:GPIbM) with gain of function and does not require ristocetin, and it can also be performed as a latex agglutination assay on a standard hemostasis analyzer [26,27]. These assays represent improved methodologies with better sensitivity to low levels of VWF and reduced variability compared to VWF:RCo. This results in more accurate diagnosis of VWD and its various types.

In addition to VWD diagnosis, these diagnostic assays can also be used to monitor therapy in VWD [12,47]. As VWD represents a loss of VWF and/or its function, and, thus, represents a hemostasis imbalance that can lead to bleeding (Figure 2), various therapies, including replacement of VWF and FVIII can be applied to correct the deficiency and treat/prevent bleeding [11,12]. Monitoring the efficacy of this therapy ensures adequate treatment and also prevents overtreatment, which can lead to thrombosis (Figure 2) [12,47].

### 5.5. Platelet Function Testing for Diagnostics

Platelets are small cells in blood that clump together at the site of an injury to produce a platelet plug to seal the injured site and prevent further bleeding. All pathology laboratories can perform platelet counts as a part of a complete blood count or profile, but only specialized laboratories are capable of performing extensive platelet function testing [48]. Historically, this was performed using a platelet aggregometer that required blood processing to isolate platelets within a fraction called platelet-rich plasma (PRP). After preparation, PRP would be challenged by various platelet aggregation agonists, and platelet aggregation was monitored. This process is time-consuming, preventing the performance of more than one or two platelet function assessments per day. PRP preparation also generated technical artifacts due to damaged or pre-activated platelets. Several innovative advances have taken place in contemporary times. First, preparation of PRP could be avoided by using a whole blood aggregometer; however, these instruments required extensive cleaning of the detection system between agonist evaluations. One recent advancement was the development of the multiplate, which uses disposable cartridges [49]. Of course, this advancement was associated with an increase in costs and laboratory waste. In addition, the ability to assess for threshold agonist responses, important to assess the degree of platelet function abnormality, was largely lost, thereby compromising diagnostic performance.

Another recent advance is the ability to perform platelet function testing on automated hemostasis analyzers [50]. Thus, several platelet function studies can be performed at once, greatly increasing test efficiency. However, preparation of PRP remains an important and time-consuming pre-test activity.

Similarly, the Optimul system permits platelet function testing to be performed in 96-well plates, and the detection of platelet aggregation using standard ELISA plate readers [51,52]. This permits effective large-scale screening, uses small volumes of blood, and can be applied in remote centers without extensive expertise in platelet function testing or access to aggregometers.

Finally, it is now also possible to undertake platelet function testing using alternate methods, including flow cytometry [53]. This also permits effective large-scale screening and can be applied in centers without extensive expertise in platelet function testing or access to aggregometers. These procedures require smaller volumes of blood, are not limited by platelet count, and do not in general require preparation of PRP.

### 5.6. Platelet Function Testing for Monitoring of Anti-Platelet Therapy

As noted previously, some patients at risk of adverse cardiovascular events, including arterial thrombosis, may be provided with various anti-platelet medications, such as aspirin, clopidogrel, and more modern agents [13]. Just as it is possible for a laboratory to use platelet function testing to diagnose a platelet function defect, laboratories can also assess the efficacy of anti-platelet medications using these assays. Although laboratories can use standard platelet function testing using an aggregometer and PRP to assess for anti-platelet medication effects, this is very time consuming, and various point-of-care test (POC) instruments can be instead used, as can the Multiplate [54]. Indeed, these POC instruments, including the Verify Now (Werfen, Barcelona, Spain), are better options for this purpose.

Another instrument that can be used for platelet function screening is the platelet function analyzer (PFA). The first version, the PFA-100 (Siemens, Malvern, PA, USA) was released in 1995, and reflected an advance on a prior instrument called the Thrombostat-4000 [55]. The PFA-100 has recently had a facelift, with the more modern PFA-200 (Siemens, Malvern, PA, USA) released in the mid-2010s, but this version is still unavailable in the USA [56]. These instruments use a small volume of whole blood (<1.0 mL) per test cartridge to assess platelet function and are sensitive to both severe platelet dysfunction and some anti-platelet medications, as well as to VWF level and function. The testing only takes around 5 min. On the other hand, this sensitivity comes at the cost of specificity, since an abnormal PFA closure time (CT) is not diagnostic of any particular defect. In our laboratory, the PFA found a home primarily as a quick screen for VWD, given its additional high sensitivity to VWF dysfunction. Thus, a normal PFA usually indicated an absence of severe VWD [56]. We have also found the PFA to be useful in monitoring VWD therapy [47].

### 5.7. Viscoelastic Testing

Although viscoelastic testing has been around for decades, it is entering a kind of renaissance, with several improved methodologies [57,58,59,60,61]. Viscoelastic testing essentially evaluates hemostasis in whole blood and can include elements of both primary and secondary hemostasis. Currently, viscoelastic testing is mostly used to define transfusion requirements to maximize replacement therapies, including, as required, such components as fresh frozen plasma (FFP), fibrinogen concentrate, or platelets, in particular in trauma and surgical procedures. The use of viscoelastic testing represents a conceptual application of precision-based medicine whereby each patient’s hemostatic phenotype is defined by the findings of the testing [61]. Some of the more modern instruments include the TEG 6s System [57], the TEG 5000 System [58], and the Quantra System [60].

### 5.8. Other Global Assays of Hemostasis

There are a large number of assays able to provide discrete information on hemostasis (e.g., see Table 1, Table 2 and Table 3). It is also possible to undertake global assessments of hemostasis. The PFA test systems mentioned earlier provide a global assessment of primary hemostasis. There are also assays that can assess overall secondary hemostasis, as well as fibrinolysis. For the former, the PT/INR and APTT assays are basic examples of assays measuring portions of secondary hemostasis. There are also assays that measure thrombin generation that represent even broader measures of secondary hemostasis [62]. As another example, the thrombin generation global thrombosis test (GTT) is an automated point-of-care technique that simulates the formation of a thrombus in whole blood under high shear flow and measures the time for occlusive thrombus formation as well as spontaneous, endogenous thrombolysis/fibrinolysis [63]. Other global assays that can help assess overall hemostasis and fibrinolysis are the “overall hemostasis potential” (OHP), the “overall coagulation potential” (OCP) and the “overall fibrinolysis potential” (OFP) tests [64,65]. However, most of these assays are currently used in research and are not as yet used in diagnostic laboratory use.

### 5.9. Monitoring Hemophilia Treatment

Hemophilia represents a loss of FVIII (hemophilia A) or FIX (hemophilia B). Classical therapy for hemophilia, therefore, required the replacement of the missing FVIII or FIX [10]. Monitoring of such a therapy is important to ensure that enough FVIII or FIX is given to prevent bleeding, but not so much, which is both wasteful of these precious resources and may lead to thrombosis if given in excess (Figure 2). In the past, this was a relatively straightforward procedure involving one-stage clotting assays for FVIII or FIX, supplemented if needed using chromogenic assays [4,25]. However, the evolution in therapy for hemophilia has been extraordinary, moving from plasma-derived to recombinant FVIII and FIX, and then from standard half-life (SHL) recombinant products to extended half-life (EHL) products to bypassing agents, FVIII mimetics, and even gene therapy. This has required a complete rethink of therapy monitoring [66,67,68,69,70,71]. First, whereas plasma-derived FVIII and FIX yielded similar results for one-stage clotting factor assays vs. chromogenic factor assays, some of the SHL recombinant products yielded different results, with either one-stage or chromogenic assays providing more or less accurate assessments of factor levels, depending on the product used. This situation has become far more complex with EHL products, and even more complex with the use of bypassing agents and FVIII mimetics. Indeed, different assays may best suit monitoring of different products, which becomes even more complex in the presence of factor inhibitors. In brief, different assays, including different chromogenic assays, some with human-origin and others with non-human-origin components, may be required for different patients and in different situations (e.g., the presence of inhibitors) [66,67,68,69,70,71].

### 5.10. Diagnosis of TTP and TTP Treatment Monitoring Innovations

TTP, or thrombotic thrombocytopenic purpura, is a life-threatening disorder caused by a deficiency of ADAMTS-13 (a disintegrin and metalloproteinase with a thrombospondin type 1 motif, member 13) activity. The function of ADAMTS-13 is to cleave VWF, in particular large VWF multimers, and, thus, reduce VWF activity. A deficiency of ADAMTS-13, in particular its absence (as in TTP) leads to an accumulation of large VWF molecules, and, thus, represents an imbalance of hemostasis (Figure 2) that leads to (micro)thrombosis. In the past, assessment of ADAMTS-13 activity required time-consuming and laborious assays, which compromised fast diagnosis of TTP, and also its monitoring during treatment [72]. ADAMTS-13 assays were improved following the further characterization of ADAMTS-13 and the development of recombinant ADAMTS-13 and VWF fragments that could be tagged with chromophores [64]. In the immediate past, these assays were developed into ELISA (enzyme-linked immunosorbent assay) methods. However, these still required hours to provide a test result. Recently, the advent of automated CLIA and FRETs (fluorescence resonance energy transfer) methods have revolutionized the diagnosis of TTP, with assays completed in half an hour [73,74,75,76]. These automated methods have also improved the monitoring of therapy, since the efficacy of treatments can be assessed in a timelier manner. The same assays used to assess ADAMTS-13 activity can also be used to assess for inhibitors of ADAMTS-13, which is especially important in diagnosing acquired or immune TTP, representing well over 90% of all TTP cases [74,77].

### 5.11. Lupus Anticoagulant Testing

LA testing reflects one of the most commonly performed procedures in specialized hemostasis laboratories. LA testing may be performed to investigate an unexpectedly prolonged APTT, or for the evaluation of patients presenting with thrombosis or pregnancy morbidity, and as a diagnostic tool for antiphospholipid syndrome (APS) [24]. In the past, testing was performed using a variety of tests, mostly using manual clot-based assays, including kaolin clotting time, and platelet neutralization. Most of the older assays are no longer performed, and instead a large variety of more modern assays, often based on snake venom activation of clotting factors, are now performed on automated hemostasis analyzers [24,78,79]; these approaches may also include global assays, such as thrombin generation [80]. Current diagnostic guidelines recommend using two tests with different principles before excluding LA in any given patient or situation, with LA-sensitive APTT and dRVVT (dilute Russell Viper venom time) assays being recommended [24]. In addition to being sensitive to LA, assays can be made more specific for LA using paired reagents, one of which has low phospholipid composition (to be LA-sensitive), while the other has a high phospholipid composition (to be relatively insensitive to LA). Thus, a sample yielding prolonged clotting times with the LA-sensitive reagent, but reduced clotting times with the LA-insensitive reagent, is considered to be ‘diagnostic’ for LA. While this testing works well in patients not under coagulation therapy, the procedures tend to fail when patients are on clinical anticoagulant therapy.

Unfortunately, the risk of assessing patients for LA whilst under clinical anticoagulant therapy is now very high, with this leading to both false-positive and false-negative LA findings [24,81]. The time course of testing is such that testing is often applied to patients who have an ‘unexpected’ prolonged APTT, or to those who have suffered a recent thrombosis. In the first case, the ‘unexpected’ prolonged APTT might itself be due to the presence of anticoagulant therapy, and in the second situation, treatment for thrombosis includes the use of anticoagulant therapy, so this may have already been applied prior to sample testing. Most clinical anticoagulants affect clotting times, including in APTT and dRVVT assays. Indeed, these anticoagulants may provide complex and difficult to interpret test patterns [Table 4]. For example, both dabigatran and rivaroxaban tend to produce test results that lead to false-positive LA patterns using the dRVVT assays, whilst apixaban use may lead to a false-negative dRVVT LA pattern [81]. In contrast, since most dRVVT reagents contain heparin neutralizers, heparin is more likely to affect APTT assays. VKA use will affect both APTT and dRVVT assays.

Fortunately, strategies have been developed to help overcome these anticoagulant interferences. As noted, most dRVVT reagents contain heparin neutralizers and so are unaffected by therapeutic levels of UH. However, APTT reagents generally do not include these heparin neutralizers, since these may alternatively be used for UH monitoring. One strategy, then, could be to replace the standard CaCl_2_ used in APTT assays with a CaCl_2_ containing a heparin neutralizer [82]. Alternate strategies include the use of methods that are relatively insensitive to anticoagulant interferences. For example, the Taipan snake venom time (TSVT) assay is insensitive to the effects of anti-FXa inhibitors, such as rivaroxaban and apixaban, as well as VKAs, but is LA-sensitive. The TSVT assay can be paired with the ecarin time assay as the LA-confirmatory test since it is insensitive to LA and to the effects of anti-FXa inhibitors and VKAs [78,79]. An alternative to overcome the anticoagulant effect of DOACs is to use DOAC neutralizers, as discussed in the next section.

### 5.12. Anticoagulant Neutralizers

As noted several times in this review, clinical anticoagulants cause prolongation in most clot-based assays. Indeed, clinicians and laboratories use several tests to monitor anticoagulant therapy (Table 4). These include the INR for monitoring VKAs, the APTT and anti-FXa assays for monitoring heparin therapy, direct thrombin inhibition (DTI) assays for dabigatran, and specific anti-FXa assays for the anti-FXa DOACs. However, the presence of these clinical anticoagulants causes unwanted interference in most of the other clot-based assays used within routine coagulation and specialized hemostasis laboratories, and sometimes also in chromogenic assays. To make the INR sensitive to VKAs, but insensitive to heparin, PT reagents tend to include heparin neutralizers (Table 4). However, APTT reagents do not contain heparin neutralizers, since many of these are used to monitor UH heparin therapy. Most dRVVT reagents also contain heparin neutralizers to enable better specificity for LA. Unfortunately, the changing landscape of anticoagulant therapy creates new and ongoing challenges for manufacturers and laboratories aiming to create hemostasis assays relatively insensitive to anticoagulant interference. Fortunately, many accomplished scientists have risen to the challenge. As mentioned above, one strategy to make the APTT insensitive to heparin, and, thus, more specific for LA, is to use a CaCl_2_ reagent that contains a heparin neutralizer [82]. DOACs are now the leading clinical anticoagulants in clinical use, and these cause complex changes to hemostasis assays (Table 4).

One very useful recent innovation, then, is the development and use of DOAC neutralizers capable of absorbing all current DOACs, and, thus, enabling hemostasis tests to be used as intended [83]. Our own experience is with a commercial product called DOAC-Stop, which can be added to DOAC-containing samples to facilitate more accurate testing for a wide range of assays, including factor assays, LA assays, and assays for APCR (activated protein C resistance) [81,84,85]. However, other commercial options are now available, and all have been shown to be effective in DOAC neutralization [81,83].

### 5.13. Harmonization and Standardization

Another innovation in hemostasis diagnostics is the process of harmonization and standardization in testing. Our own experience in this process includes harmonization and standardization within the EQA process, and also as applied to a large network of 60 laboratories performing routine coagulation tests with over 85 hemostasis instruments [86]. Using this process, our large network can now standardize, to single lots of routine reagents (e.g., PT/INR and APTT), single MNPT and ISI values for INR across all laboratories using the same PT reagent, common standardized reference ranges for all tests performed in the network using the same reagents and instrument class, as well as common therapeutic ranges for anticoagulant monitoring, including the APTT therapeutic range for UH. This process enables huge time and cost savings, as well as numerous other efficiencies across the entire network. Additional examples of harmonization and standardization initiatives are detailed elsewhere, and include the external quality assessment process [87,88].

## 6. Future Innovative Diagnostic Solutions in Hemostasis

Of course, no one has a magical crystal ball able to accurately identify future innovations that will provide improved diagnostic solutions in hemostasis. Nevertheless, we can conjecture, based on past experience.

### 6.1. Anticoagualant Neutralizers

For DOAC neutralization, the current process requires adding the product (typically activated charcoal) to the sample containing the DOAC, and then centrifuging out the material with the DOAC absorbed out of the sample. Then, the ‘cleaned’ sample can be used for diagnostic testing. Although this process works, it requires additional time and effort, and for a busy lab, such as ours, performing in excess of 100 LA tests a week, it could be difficult to enact uniformly. Centrifugation is required because the activated charcoal product would itself interfere with the clot-detection optics of many automated hemostasis analyzers. Similarly, the product cannot be added to current reagents, since this would prevent subsequent centrifugation to remove the DOAC neutralizer. However, we have recently shown in a proof-of-concept paper that a liquid form of DOAC-Stop can be used without centrifugation for mechanical clot-detection instruments [83]. This may lead to the development of DOAC neutralizers added to select hemostasis reagents for immediate use in select mechanical test systems, and without the need for an extra centrifugation step.

### 6.2. Emerging Anticoagulants

We would also be naïve to think that we have seen the last clinical anticoagulant to be developed. The DOACs represent the current state of the art in clinical anticoagulant therapy, but there are other anticoagulants in development; for example, the so-called anti-FXI agents [89,90]. As such anticoagulants are developed and then deployed, manufacturers and laboratories will need to consider methods to monitor these agents (if required) and also to overcome unwanted interference in diagnostic assays (as required). Similarly, whilst aspirin reflects a classical antithrombotic, the anti-P2Y12 inhibitors were developed to improve the overall efficacy of antithrombotic therapies [91]. These include the agents clopidogrel, ticlopidine, ticagrelor, prasugrel, and cangrelor. Clinicians may want to periodically monitor the efficacy of these agents with platelet function assays, and perhaps some emerging POC methods. These evaluations could possibly take place in the cardiologists’ rooms and would ideally require only a finger-prick sample.

### 6.3. Emerging Procoagulants

Similarly, there are a plethora of procoagulant or hemostasis rebalancing agents under development [10,11,66,67,68,69,70,71]. These could be applied to a wide range of patients at risk of bleeding, either because they have a deficiency (e.g., of FVIII, FIX, or VWF) or because they have been hemostatically challenged (e.g., trauma, menstruation, or surgery). Laboratories may need to monitor the efficacy of such treatments in select situations. Given the diverse nature of the products under development, broad or global assays, such as thrombin generation, will perhaps finally find a place in most hemostasis laboratories.

### 6.4. Emerging Diagnostic Advancements

Platelet function testing typically involves the assessment of blood or PRP using discrete platelet agonists. These tests typically also require samples containing normal platelet counts. It would be diagnostically useful to develop additional assays that have less constraints and better reflect in vivo physiology [92,93,94]. For example, most test systems do not use flow shear to assess platelet function, and the involvement of the endothelium is absent. We look forward to the test systems of the future that will enable this.

Finally, why settle on a process that restricts assessment to individual hemostasis test components or platelet function? We can envisage the development of more global test systems to supplement current global test systems, for example thrombin generation for secondary hemostasis and the PFA for primary hemostasis. As our understanding of hemostasis pathways develops, so will models for mimicking hemostasis and thrombosis, and, subsequently, instruments and tests that will model and mimic hemostasis and thrombosis, perhaps using in vitro flow-based assays [95]. Moreover, the use of additional new platelet transcriptome methods can improve our understanding of platelet reactivity, for example its contribution to peripheral artery disease [96].

## 7. Conclusions

The field of hemostasis testing has witnessed significant innovation over the past decades, driven by the need for more accurate, efficient, and comprehensive testing methods. From the evolution of routine coagulation assays to the development of specialized tests, these innovations have greatly enhanced our ability to diagnose and manage bleeding and thrombotic disorders. These advancements not only improve diagnostic accuracy, but also facilitate better patient management by enabling tailored therapeutic approaches. We have tried to capture some of that innovation in our review. It is inevitable that such innovation will continue in the future. The future of this field also lies in the harmonization and standardization of testing protocols, the creation of more global and physiologically relevant assays, and the seamless incorporation of these innovations into clinical practice. We give praise to those accomplished people who are working to innovate and ultimately improve the diagnostic landscape that is hemostasis testing. We also await with interest the involvement of artificial intelligence (AI) in future innovations [97] to ensure more precise diagnostics, optimized treatments, and, ultimately, better patient outcomes in managing hemostatic disorders.

## Figures and Tables

**Table 1:** Routine coagulation assays. These tests will be available in most hemostasis testing laboratories *.

Test Abbreviation	Test	What the Test Measures	What the Test Is Used For	What Else Is the Test Sensitive to?
PT **	Prothrombin time	Tissue factor (TF) (also called extrinsic) pathway plus common pathway	Assessment of factor deficiency (I, II, V, VII, X). Monitoring of VKAs (e.g., warfarin) therapy (typically as the INR), and screening for DIC	Various anticoagulants (e.g., UH in excess to heparin neutralizer capacity, DOACs)
INR	International normalized ratio	Same as PT, but reflective of a normalized ratio	Used to monitor patients on VKA therapy	Same as PT
APTT	Activated partial thromboplastin time	Contact factor (also called intrinsic) pathway plus common pathway	Assessment of factor deficiency (I, II, V, VIII, IX, X, XI, XII), monitoring of UH therapy, and screening for DIC	Various anticoagulants (e.g., DOACs, LMWH)
TT	Thrombin Time	Measure of fibrinogen clotting activity	Screen for fibrinogen deficiency. Screen for UH and other anti-II agents (e.g., dabigatran)	Various anticoagulants (e.g., lepirudin, bivalirudin, LMWH)
D-D	D-dimer	The fibrin degradation product called D-dimer	Screen for venous thrombosis (e.g., deep vein thrombosis [DVT]; pulmonary thrombosis [PE]). Screen for DIC	Depending on the antibody used in assay, potentially variously sensitive to other fibrin or fibrinogen degradation products
Fib or FGN	Fibrinogen	Fibrinogen level (fibrinogen is the major coagulation protein)	Assessment of congenital or acquired fibrinogen deficiencies or abnormalities. Screen for DIC	Some assays may be affected by very high levels of some anticoagulants (e.g., UFH, dabigatran)

* See also Figure 1. ** The PT can be analyzed using the Owren or Quick method, with the Owren method being sensitive to factors II, VII, and X, and the Quick method sensitive to factors I, II, V, VII, and X. The Owren PT assay is predominantly used in some countries, such as Nordic countries, but the Quick method is more widely used, including within Australia and North America. DIC, disseminated intravascular coagulation; DOACs, direct oral anticoagulants; LMWH, low molecular weight heparin; UH, unfractionated heparin; VKA, vitamin K antagonists.

**Table 2:** Specialized hemostasis assays. These tests will be selectively available in different hemostasis testing laboratories *.

Test Abbreviation	Test	What the Test Measures	What the Test Is Used For	What Else Is the Test Sensitive to?
AT	Antithrombin	Antithrombin level or activity	Quantitation of antithrombin activity	Depending on how the assay is performed (i.e., based on anti-FXa or anti-FIIa), it may be sensitive to various anticoagulants (e.g., DOACs).
PC	Protein C	Protein C level or activity	Quantitation of Protein C activity	Clot-based assays may be affected by various anticoagulants, including DOACs and VKAs.
PS	Protein S	Protein S level or activity	Quantitation of Protein S level or activity	Clot-based assays may be affected by various anticoagulants, including DOACs and VKAs.
LA	Lupus anticoagulant	Presence or absence of LA	To exclude/identify LA for diagnosis of APS or as a cause of APTT prolongation	Various anticoagulants depending on assays/reagents employed.
Anti-Xa or anti-FXa	Anti-factor Xa	Level of various anticoagulants depending on test set up	To quantify levels of UH, LMWH, direct and indirect anti-FXa agents (e.g., apixaban, rivaroxaban, edoxaban, fondaparinux)	Each ‘specific’ anti-FXa assay is variously sensitive to other anti-FXa agents.
DTI or dTT	Direct thrombin inhibitor or dilute thrombin time	Level of various anticoagulants depending on test set up	To quantify levels of anti-FIIa agents (e.g., dabigatran)	Each ‘specific’ anti-FIIa assay potentially sensitive to other anti-FIIa agents.
FII, FV, FVII, FVIII, FIX, FXI, and FXII	Factors II, V, VII, VIII, IX, XI, and XII	Level and activity of these clotting factors	To quantify these factor levels	All clot-based assays variably sensitive to various clinical anticoagulants.
FXII	Factor XIII	Level and activity of FXIII	Quantitation of FXIII	May depend on the assay.
VWF	von Willebrand factor	Level and activity of VWF	To quantify VWF and its various activities	Different functional assays tend to be ‘specific’ for a particular VWF activity.
ADAMTS-13	ADAMTS-13	Level and activity of ADAMTS-13	To quantify ADAMTS-13 activity	May depend on the assay.
PFS	Platelet function studies	Platelet activity	To quality platelet activity or diagnose platelet dysfunction	Depends on the assay.

* ADAMTS-13, a disintegrin and metalloproteinase with a thrombospondin type 1 motif, member 13; APS, antiphospholipid syndrome; LMWH, low molecular weight heparin; UH, unfractionated heparin.

**Table 3:** An evolution in VWF testing in the diagnosis of VWD, and in monitoring its therapy. These tests will be selectively available in different hemostasis testing laboratories *.

Test Abbreviation	Test	What the Test Measures	What the Test Is Used For	How Is the Test Performed?
VWF:Ag	VWF antigen	Level of VWF	Quantitation of VWF level	Usually LIA or ELISA; sometimes CLIA
RIPA	Ristocetin induced platelet agglutination/ aggregation	Activity of VWF binding to GPIb	Qualification of VWF GPIb binding activity	Platelet agglutination assay, usually on a platelet aggregometer
VWF:RCo	VWF ristocetin cofactor	Activity of VWF binding to GPIb	Quantitation of VWF GPIb binding activity	Platelet agglutination assay, usually on an automated hemostasis analyzer, sometimes on a platelet aggregometer
VWF:CB	VWF collagen binding	Activity of VWF binding to collagen (a matrix protein exposed by vascular damage)	Quantitation of VWF collagen binding activity	Usually ELISA; sometimes CLIA
VWFpp	VWF propeptide	Level of VWF propeptide	To quantify VWF propeptide as a marker of VWF clearance	ELISA
VWF:FVIIIB	VWF factor VIII binding	Activity of VWF binding to FVIII	Quantitation of VWF FVIII binding activity	ELISA
VWF:GPIbR	VWF GPIb recombinant	Activity of VWF binding to recombinant GPIb	Quantitation of VWF GPIb binding activity	Usually latex agglutination assay on automated hemostasis analyzer; sometimes CLIA
VWF:GPIbM	VWF GPIb (recombinant) mutant	Activity of VWF binding to recombinant mutated GPIb	Quantitation of VWF GPIb binding activity	Usually latex agglutination assay on automated hemostasis analyzer; sometimes ELISA

* LIA, latex immunoassay; CLIA, chemiluminescence immunoassay; ELISA, enzyme-linked immunosorbent assay; VWF, von Willebrand factor; VWD, von Willebrand disease; CB, collagen binding; RCo, ristocetin cofactor; GPIb, glycoprotein Ib (the platelet VWF receptor); R, recombinant; M, mutant.

**Table 4:** Interference of anticoagulants vs. laboratory monitoring of anticoagulants vs. in vitro neutralization of anticoagulants *.

		Anti-FXa DOACs	Anti-FIIa DOACs (Dabigatran)	VKAs	Heparins (UH/LMWH)
Test/Parameter	Monitor or measure with:	Specific anti-FXa assays	Specific anti-FIIa assay (e.g., direct thrombin inhibitor [DTI) assay. Ecarin-based assays	PT/INR	APTT, anti-FXa assay
	Neutralize with:	Activated charcoal (e.g., DOAC-Stop)	Activated charcoal (e.g., DOAC-Stop)	- (mixing studies)	polybrene, hepzyme
	PT/INR depends on:	-/↑/↑↑ DOAC, [DOAC], reagent	-/↑ [DOAC], reagent	↑/↑↑/↑↑↑ [VKA], reagent	-/↑ heparin type, [heparin], presence of neutralizers
	APTT depends on:	-/↑ DOAC, [DOAC], reagent	↑/↑↑ [DOAC], reagent	↑/↑↑ [VKA], reagent	↑/↑↑/↑↑↑ heparin type, [heparin], reagent
	TT depends on:	-	↑↑↑	-	↑/↑↑/↑↑↑ heparin type, [heparin], reagent
	D-D	-	-	-	-
	Fib depends on:	-/↓ [DOAC], reagent	-/↓ [DOAC], reagent	-	-/↓ heparin type, [heparin], reagent
	Anti-FXa assays depend on:	↑/↑↑/↑↑↑ [DOAC]	-	-	↑/↑↑/↑↑↑ [heparin]
	Factor assays depend on:	↓/↓↓ [DOAC]	↓/↓↓ [DOAC]	↓/↓↓ Factor type, [VKA]	-(/↓) heparin type, [heparin]
	PC and PS depend on:	-/↑ [DOAC], reagent	-/↑ [DOAC], reagent	↓/↓↓ [VKA]	-(/↓) heparin type, [heparin], reagent
	AT depends on:	-/↑ [DOAC], reagent	-/↑ [DOAC], reagent	-	-/↓ heparin type, [heparin], reagent
	APCR depends on:	-/↑ [DOAC], reagent	-/↑ [DOAC], reagent	-/↑ [VKA], reagent	-/↑ heparin type, [heparin], reagent
	LA depends on:	-/↓/↑/↑↑ DOAC type/[DOAC], reagent	↑/↑↑ [DOAC], reagent	-/↓/↑/↑↑ [VKA], reagent	-/↑ heparin type, [heparin], reagent, presence of heparin neutralizers
	VWF, platelet function	-	-	-	-
	TGA depends on:	↓/↓↓/↓↓↓ DOAC type/[DOAC], reagent	↓/↓↓/↓↓↓ [DOAC], reagent	↓/↓↓/↓↓↓ [VKA], reagent	↓/↓↓/↓↓↓ heparin type, [heparin], reagent
	VEA	↑/↑↑ [DOAC], reagent, system	↑/↑↑ [DOAC], reagent, system	-/↑ [VKA], reagent, system	-/↑ heparin type, [heparin], reagent, system

* Anti-FXa DOACs include apixaban, rivaroxaban, and endoxaban. Anti-FIIa DOACs currently comprise dabigatran. Up arrows (↑) indicate an increase, down arrows (↓) indicate a decrease, and dashes (-) indicates a status that is not expected to change. Number of arrows indicate the extent of change (one arrow indicates a low effect, two arrows indicate a moderate effect, and three arrows indicate a high effect). Abbreviations: APCR, activated protein C resistance; APTT, activated partial thromboplastin time; AT, antithrombin; D-D, D-dimer; DOACs, direct oral anticoagulants; Fib, fibrinogen; LA, lupus anticoagulant; VKAs, vitamin K antagonists; LMWH, low molecular weight heparin; UH, unfractionated heparin; PC, protein C; PS, protein S; PT/INR, PT, prothrombin time; TGA, thrombin generation assay; INR, international normalized ratio; TT, thrombin time; [DOAC], DOAC level or concentration; [VKA], VKA level; [heparin], heparin level or concentration; VEA, viscoelastic assays; VWF, von Willebrand factor.

## Data Availability

Not applicable; no new data were created in this review.
